# The therapeutic potential of targeting the EGFR family in epithelial ovarian cancer

**DOI:** 10.1038/bjc.2011.62

**Published:** 2011-03-01

**Authors:** Q Sheng, J Liu

**Affiliations:** 1 Novartis Oncology Translational Research, Novartis Pharmaceuticals, 250 Massachusetts Avenue, Cambridge, MA 02139, USA; 2 Department of Medical Oncology, Dana-Farber Cancer Institute, 44 Binney Street, Boston, MA 02115, USA

**Keywords:** EGFR family, ErbB3, ovarian cancer

## Abstract

The epithelial growth factor receptor (EGFR) family of receptor tyrosine kinases has been reported to have an active role in a number of malignancies. Amplifications and overexpression of various EGFR family members, including EGFR, Her2, and ErbB3, have been reported in epithelial ovarian cancer. Although anti-EGFR-targeted therapy has shown limited clinical activity in ovarian cancer to date, a recent report suggests that activation of ErbB3, one of the members of the EGFR family, may support the growth and proliferation of ovarian cancer cells and that ErbB3 may therefore serve as a potential therapeutic target in this disease. Here, we review the EGFR family and the clinical experience with anti-EGFR family member-directed therapies in ovarian cancer to date.

The epithelial growth factor receptor (EGFR; also known as Her or ErbB) family of receptor tyrosine kinases has been shown to have oncogenic roles in a number of human cancers. Epithelial growth factor receptor family-directed therapies have demonstrated significant clinical activity in EGFR mutant lung cancers and Her2-amplified breast cancers. Recent reports have suggested that members of the EGFR family, such as ErbB3, may have a role in supporting the growth and proliferation of human ovarian cancer cells. Epithelial ovarian cancer remains a leading cause of death in women, with an estimated 204 000 cases and 125 000 deaths worldwide (Parkin *et al*, 2005). Here, we review the reported biology and clinical experience with EGFR family-directed therapies in epithelial ovarian cancer.

## Structure and signalling of epithelial growth factor receptor family members

The EGFR family of receptor tyrosine kinases consists of four closely related family members: EGFR (Her1), ErbB2 (Her2), ErbB3 (Her3), and ErbB4 (Her4; Yarden, 2001). Throughout this review, these kinases shall be referred to as EGFR, Her2, ErbB3, and ErbB4. These cytoplasmic membrane-bound receptors share a common extracellular ligand-binding domain and a single transmembrane domain, followed by an intracellular tyrosine kinase domain and a C-terminal non-catalytic signalling tail. Signalling through these receptors is typically mediated by homodimerisation or heterodimisation with other family members. With the exception of Her2, the extracellular domains of the EGFR family members prevent the formation of stable heterodimers or homodimers in the absence of the ligand (Ogiso *et al*, 2002). Binding of the ligand with the extracellular domain of its corresponding receptor induces a structural reconfiguration of the receptor, promoting exposure of the otherwise tethered dimerisation domain (Ogiso *et al*, 2002). This, in turn, leads to stabilisation of receptor dimers. Dimerisation results in asymmetric kinase activation, transphosphorylation, and induction of downstream signalling pathways. The EGFR family members can also be activated by ligand-independent activation (Siwak *et al*, 2010). These mechanisms include activation by unphysiological stimuli (e.g., oxidative stress, UV, and *γ*-irradiation), by other receptor tyrosine kinases (notably MET, insulin-like growth factor-1 receptor, or tyrosine kinase receptor B), or by G protein-coupled receptors and adhesion proteins.

The importance of dimerisation in EGFR family signalling is best illustrated by Her2 and ErbB3. Her2 is the preferred dimerisation partner for all of the EGFR family members (Tzahar *et al*, 1996). However, although Her2 has the strongest kinase activity among the family members, there is no known ligand to this receptor. Consequently, activation of Her2 is dependent upon dimerisation with other family members. In contrast, ErbB3 can be bound by at least two known ligands, but lacks intrinsic kinase activity and is therefore dependent upon heterodimerisation to phosphorylate its signalling tail and mediate downstream signalling effects. Interestingly, these so called ‘deaf and dumb’ members of the EGFR family form the most potent transforming unit among all of the possible dimerisation units (Tzahar *et al*, 1996).

Multiple ligands are known to bind to the EGFR family. Some of these ligands bind specifically to EGFR (EGF, TGF-*α*, and amphiregulin) or ErbB4 (neuregulin 3, neuregulin 4, and tomoregulin), whereas others have dual specificity (e.g., *β*-cellulin, epiregulin, and heparin-binding EGF-like growth factor for both EGFR and ErbB4, and neuregulins 1 and 2 for both ErbB3 and ErbB4; Normanno *et al*, 2006). Various expression levels of these ligands have been detected in both ovarian cancer cell lines and primary ovarian tumour samples (Lafky *et al*, 2008).

Activated EGFR family members recruit various adaptors and signalling molecules through the phosphorylated cytoplasmic domain, which further leads to activation of a variety of downstream signalling pathways (reviewed by Normanno *et al*, 2006). All of the EGFR family members activate the extracellular signal-regulated kinase (Erk)1/2 via recruitment of Grb2 or Shc adaptors. Activation of Erk1/2 has an important role in EGFR-stimulated cell proliferation. Activation of another important signalling pathway for cell proliferation and survival, the phosphatidylinositol 3-kinase (PI3K)/Akt pathway, however, differs between the EGFR family members. Whereas ErbB3 and ErbB4 are capable of binding the p85 regulatory subunit of PI3K directly through their putative p85 binding sites (tyrosine-X-X-methionine), EGFR and Her2 bind indirectly to p85 through adaptors or through heterodimerisation with ErbB3 or ErbB4. In addition to Erk1/2 and PI3K activation, phosphorylated EGFR family members can also activate a collection of transcription factors such as c-fos, c-jun, c-myc, signal transducer and activator of transcription, NF-*κ*B, zinc-finger transcription factor, and Ets family members.

## Epithelial growth factor receptor/Her2 in ovarian cancer

A recent review by Siwak *et al* (2010) provides a comprehensive update on the role of EGFR in ovarian cancer and the experience with EGFR-targeted therapies in this disease. Epithelial growth factor receptor amplification and activating mutations have been reported in a small percentage of ovarian cancer cases (4–22% and <4%, respectively). The EGFR overexpression rate varies from 9–62%, depending on the antibody, the assay, and the cutoff standard. Increased EGFR expression has been correlated with poorer patient outcomes. Several small molecule inhibitors that block EGFR kinase activity (e.g., gefitinib and erlotinib) have been explored in the clinical setting. There are two phase II clinical trials on gefitinib (Iressa or ZD1839) as a single agent in treating platinum-refractory or -resistant ovarian cancer. No complete response was observed in either trial. In one trial, 37% of the patients had stable disease for over 2 months while none of the patients had a partial or complete response. In the second trial, 4 out of 27 patients had progression-free disease for over 6 months and one of these four patients had an objective response. Of note, tumour samples from the patient who experienced a partial response demonstrated a mutation in the EGFR kinase domain. In this trial, EGFR expression was also evaluated by immunohistochemistry, and a correlation between EGFR expression and longer progression-free and overall survival was suggested (*P*=0.008 and *P*=0.08, respectively). A phase II study combining gefitinib and tamoxifen also demonstrated limited activity in patients with platinum-refractory or -resistant ovarian cancer. Of note, these trials were performed in an unselected patient population. Erlotinib (Tarceva, OSI Pharmaceuticals, Long Island, NY, USA), another small molecule inhibitor of EGFR, demonstrated a 6% partial response rate in a multicentre phase II trial with EGFR-positive ovarian cancer patients with taxane- and/or platinum-refractory or -resistant disease.

Trials of EGFR-targeted agents with cytotoxic chemotherapy or other targeted therapies have also been conducted. In a separate phase I trial, when erlotinib was administered in conjunction with docetaxel and carboplatin in 48 patients, a slightly lower response rate was reported than in a historical control with docetaxel and carboplatin alone (52 *vs* 59% Siwak *et al*, 2010). More recently, a phase II study of 56 patients demonstrated no improvement in pathological CR rates compared with historical experience with erlotinib added to carboplatin and paclitaxel in the first-line therapy for ovarian cancer (Blank *et al*, 2010). Tumour specimens were analysed for EGFR amplification in 20 patients, but no statistically significant correlation was observed between amplification status and response. An additional study comparing the combination of erlotinib and the anti-angiogenic agent bevacizumab also did not show any improvement compared with bevacizumab alone. Similarly, vandetanib, a small molecule inhibitor of VEGFR and EGFR signalling, did not demonstrate significant clinical benefit in recurrent ovarian cancer (Annunziata *et al*, 2010). In addition to small molecule inhibitors, monoclonal antibodies that bind directly to the extracellular domain of EGFR to block EGFR activation or reduce surface EGFR levels have also been tested in clinic. The clinical activity of these therapeutic antibodies, such as cetuximab (Erbitux, ImClone LLC, Bridgewater, NJ, USA) and matuzumab (EMD7200), in ovarian cancer appears to be very limited (Siwak *et al*, 2010).

Data regarding Her2 overexpression and its association with prognosis in ovarian cancer are controversial. Early studies suggested that Her2 overexpression in ovarian cancer was a frequent event; however, most recent studies using techniques validated in breast cancer suggest that Her2 overexpression and amplification frequency in ovarian cancer is, in fact, a much rarer event (Farley *et al*, 2009). Overexpression of Her2 has been associated with a worse prognosis in some studies but not others. This discrepancy may be in part explained by differences in the criteria and methods used to assess Her2 overexpression. Clinically, anti-Her2-directed therapies have not demonstrated significant promise as therapeutic agents in ovarian cancer. A phase II trial with trastuzumab (Herceptin, Genentech, Inc., South San Francisco, CA, USA), a humanised anti-Her2 monoclonal antibody, which has been used to successfully treat Her2-overexpressing breast cancer, reported a low (7%) partial response rate with only a 2-month progression-free interval in recurrent ovarian cancers overexpressing Her2 (Bookman *et al*, 2003).

Reagents targeting multiple EGFR family members simultaneously have also been tested for their clinical activity in ovarian cancer. In a phase II multicenter open-label trial, CI-1033 (Canertinib, Pfizer, Inc., New York City, NY, USA), an irreversible pan-EGFR family inhibitor, was shown to have no activity in unscreened patients who have failed previous platinum-based chemotherapy (Campos *et al*, 2005). In this trial, the expression of EGFR family members was also examined by IHC in archival tumours and there was no association of EGFR family member expression level and clinical outcome. A phase I trial combining carboplatin and lapatinib (Tykerb, Tyverb, Glaxo SmithKline, plc, London, UK), an EGFR-Her2 dual inhibitor, was performed in unscreened patients with platinum-sensitive recurrent ovarian cancer (Kimball *et al*, 2008). Three out of eleven (27%) patients had a partial response and three patients (27%) had stable disease. However, given significant non-dose limiting toxicities, including grade 3 neutropenia and grade 4 thrombocytopenia, significant treatment delays, and minimum clinical benefit, the authors did not feel that this treatment regimen could be recommended for further exploration.

## ErbB3 in ovarian cancer

ErbB3 has been reported to be amplified (Tsuda *et al*, 2004) and overexpressed in epithelial ovarian cancers (Simpson *et al*, 1995; Tsuda *et al*, 2004; Tanner *et al*, 2006; Lafky *et al*, 2008; Sheng *et al*, 2010). In certain studies, overexpression of ErbB3 has been reported to correlate with poorer overall prognosis (Tanner *et al*, 2006). In addition to full-length ErbB3, several truncated ErbB3 isoforms composed of the extracellular domains of the protein have also been previously described in ovarian cancer cell lines, but the functional significance of these remains unclear (Lee and Maihle, 1998).

ErbB3 has been recently characterised as having a significant role in mediating resistance to EGFR- and Her2-directed therapies in other solid malignancies. Sergina *et al* (2007) demonstrated that in gefitinib-resistant Her2-overexpressing breast cancer cell lines, ErbB3 activation is increased, potentially through increased localisation of ErbB3 to the cytoplasmic membrane. In a separate report, Engelman *et al* (2007) demonstrated that ErbB3 activation by MET amplification could overcome resistance to gefitinib in EGFR-mutant non-small-cell lung cancer cell lines. Most recently, inhibition of AKT has been demonstrated to upregulate ErbB3 expression and phosphorylation, suggesting that ErbB3 may also have a role in mediating resistance to PI3K/AKT pathway inhibitors (Chandarlapaty *et al*, 2011).

A humanised monoclonal antibody that binds to the extracellular domain of Her2 and blocks its heterodimerisation with ErbB3 and other EGFR family members, pertuzumab (2C4), has been explored in clinical trials in ovarian cancer. As a single agent, pertuzumab treatment demonstrated only a 4.3% partial response in platinum-refractory and -resistant patients. Her2 phosphorylation (pHer2) status in 28 of these patients with biopsiable disease (out of a total of 55 patients) was assayed, with eight tumours demonstrating positivity for pHer2 by an ELISA assay. ErbB3 status was not assessed in this trial. The median progression-free survival in the eight pHer2+ patients was 20.9 weeks as compared with 5.8 weeks in the pHer2− patients (*P*=0.14), and the possibility was raised that response to pertuzumab might be linked to the presence of pHer2 as opposed to overexpression of the protein (Gordon *et al*, 2006). A clinical trial has also been conducted exploring the combination of pertuzumab with gemcitabine *vs* gemcitabine alone (Makhija *et al*, 2010). In this trial, no significant difference was seen in the outcome between the two arms. However, a subset analysis demonstrated a potential benefit in progression-free survival to gemcitabine plus pertuzumab in patients with low ErbB3 expression by mRNA (hazard ratio 0.32; 95% CI 0.17–0.59; *P*=0.0002).

More recently, signalling through the ErbB3 pathway has been reported to correlate with expression of its natural ligand, neuregulin-1 (NRG1) in both ovarian cancer cell lines and in primary human ovarian cancer cells (Sheng *et al*, 2010). Expression of NRG1 has been observed in 30–83% of ovarian carcinomas (Gilmour *et al*, 2002; Sheng *et al*, 2010). Furthermore, in a subset of ovarian cancer cells that display an NRG1-expressing/phosphorylated-ErbB3 phenotype, depletion of ErbB3 by RNA interference (RNAi) could result in anti-proliferative effects *in vitro*. In a mouse model of human ovarian cancer, depletion of ErbB3 by RNAi reduced tumour growth and prolonged mouse survival, while treatment with a monoclonal anti-ErbB3 antibody (MM-121, Merrimack Pharmaceuticals, Cambridge, MA, USA) also resulted in inhibition of tumour progression (Sheng *et al*, 2010). Clinically applicable therapeutic anti-ErbB3 antibodies, including MM-121 and U3-1287 (Amgen, Thousand Oaks, CA, USA), are currently being tested in phase I trials in solid tumours (http://www.clinicaltrials.gov). Their potential activity as single agents or in combination with additional cytotoxic or biological therapies in ovarian and other cancers is currently an area of active interest and exploration.

## ErbB4 in ovarian cancer

Among different EGFR family members, the role of ErbB4 in cancer is probably the least understood. Conflicting reports regarding the potential transforming activity of ErbB4 have been published, which may partly reflect the unique mechanism of ErbB4 signalling. Unlike the other EGFR family members, ErbB4 is spliced into multiple isoforms, some of which are further processed by tumour necrosis factor-*α* converting enzyme and *γ*-secretase to generate a soluble ErbB4 intracellular domain that has a BH3 homologous region that can distribute to both the nucleus and the cytoplasm (reviewed by Jones, 2008). Nuclear-localised ErbB4 intracellular domain may act as a transcription coactivator whereas the cytoplasmic/mitochondria-localised ErbB4 intracellular domain may act as a BH3-only protein and induce apoptosis in tumour cells (Jones, 2008). In breast cancer, cytoplasmic but not nuclear localisation of the ErbB4 intracellular domain independently predicted for improved patient survival (Jones, 2008). Clinically, ErbB4 somatic mutations have been detected in 19% of melanoma patients (Prickett *et al*, 2009) and in a variety of other cancer types although at a much lower frequency (1–5% reviewed by Rudloff and Samuels, 2010). *In vitro* depletion of ErbB4 in some melanoma cell lines expressing mutated ErbB4 inhibited proliferation, suggesting that mutated ErbB4 may be an addicting oncogene in these particular cells (Prickett *et al*, 2009).

ErbB4 is weakly expressed in adult ovarian surface epithelium (Srinivasan *et al*, 1998). Although early studies suggested that ErbB4 expression is either absent or decreased in some ovarian cancers when compared with normal ovarian tissues (Scoccia *et al*, 1998; Srinivasan *et al*, 1998), recent investigations using either IHC, RT–PCR, or western blotting techniques have demonstrated the presence of ErbB4 in a high percentage of ovarian tumours (Gilmour *et al*, 2001; Pejovic *et al*, 2009), and RT–PCR has detected at least four different ErbB4 splice variants in both established and primary ovarian cancer cell lines (Gilmour *et al*, 2001). Cytoplasmic staining of ErbB4 was detected in 89–93% of 53 unselected ovarian cancer tumours using two different anti-ErbB4 antibodies directed to either the cytoplasmic or the extracellular domain of ErbB4 (Gilmour *et al*, 2001). In a more recent study, as many as 96% of tumours (195 of 202) had cytoplasmic, nuclear, and/or membranous ErbB4 staining (Pejovic *et al*, 2009). Gilmour *et al* (2001) reported that tumours of serous histology tend to express higher levels of ErbB4 than that of the endometrioid subtype, and Steffensen *et al* (2008) found that ErbB4 expression is significantly higher in epithelial ovarian cancer tumours as compared with borderline/benign ovarian tumours or normal ovaries. Somatic mutations in the intronic regions of ErbB4 have been detected in ovarian cancers; however, no mutations similar to that found in melanoma have been reported thus far (Pejovic *et al*, 2009).

The clinical significance of ErbB4 in ovarian cancer is currently unknown. Interestingly, established ovarian cancer cell lines that express high ErbB4 protein level have all been derived from platinum-refractory tumours, raising the possibility that ErbB4 expression may associate with the development of platinum resistance (Gilmour *et al*, 2001). On the other hand, an ErbB4 antibody that blocks ErbB4 and NRG1 interaction appeared to have a stimulatory effect in some of the ovarian cancer cell lines tested, suggesting a possible role for ErbB4 in growth inhibition in these cells (Gilmour *et al*, 2001). Furthermore, higher ErbB4 expression has been reported to associate with improved disease-free survival in ovarian cancers (Pejovic *et al*, 2009). Together, these observations suggest that the role of ErbB4 in ovarian tumours may be complicated and, to date, its function in both early ovarian cancer development and late-stage disease remains undefined.

## Conclusion

The EGFR family of receptor tyrosine kinases remains of significant interest in ovarian cancers. A summary of the characteristics of this family is found in Table 1. Amplifications, mutations, and overexpression of EGFR family members have been described in epithelial ovarian cancer, and pre-clinical data have suggested that interfering with the signalling activity of these pathways in ovarian cancer cells can result in antitumour activity. Although EGFR- and Her2-directed therapies have yielded disappointing clinical results to date, recent reports regarding the role of other members of this receptor tyrosine kinase family, such as ErbB3 and ErbB4, in ovarian cancer suggest that new approaches towards targeting the EGFR family merit further exploration.

## Figures and Tables

**Table 1:** Summary of EGFR family members

**EGFR family member**	**Ligand(s)**	**Validated clinical target in non-ovarian cancers**	**Characteristics in ovarian cancer**
EGFR	EGF	Non-small-cell	4–22% Amplification
	TGF-*α*	Lung cancer	<4% Mutation
	Amphiregulin	Colorectal	
	*β-*cellulin	Head and neck	
	Epiregulin	Pancreatic	
	HB-EGF		
Her2	None	Breast	Rare (5–10%) amplification/overexpression
ErbB3	Neuregulin 1		Expression/amplification in
	Neuregulin 2		3–90%
ErbB4	Neuregulin 1		Expression by IHC in
	Neuregulin 2		89–96%
	Neuregulin 3		
	Neuregulin 4		
	Tomoregulin		
	*β*-cellulin		
	Epiregulin		
	HB-EGF		

Abbreviations: EGFR=epithelial growth factor receptor; HB-EGF=heparin-binding EGF-like growth factor; IHC=immunohistochemistry; TGF-*α*=transforming growth factor-*α*.
